# Markov blankets, information geometry and stochastic thermodynamics

**DOI:** 10.1098/rsta.2019.0159

**Published:** 2019-12-23

**Authors:** Thomas Parr, Lancelot Da Costa, Karl Friston

**Affiliations:** Wellcome Centre for Human Neuroimaging, Institute of Neurology, University College London, London WC1N 3AR, UK

**Keywords:** thermodynamics, information geometry, variational inference, Bayesian, Markov blanket

## Abstract

This paper considers the relationship between thermodynamics, information and inference. In particular, it explores the thermodynamic concomitants of belief updating, under a variational (free energy) principle for self-organization. In brief, any (weakly mixing) random dynamical system that possesses a Markov blanket—i.e. a separation of internal and external states—is equipped with an information geometry. This means that internal states parametrize a probability density over external states. Furthermore, at non-equilibrium steady-state, the flow of internal states can be construed as a gradient flow on a quantity known in statistics as Bayesian model evidence. In short, there is a natural Bayesian mechanics for any system that possesses a Markov blanket. Crucially, this means that there is an explicit link between the inference performed by internal states and their energetics—as characterized by their stochastic thermodynamics.

This article is part of the theme issue ‘Harmonizing energy-autonomous computing and intelligence’.

## 1. Introduction

Any object of study must, implicitly or explicitly, be separated from the rest of the universe. This implies a boundary that separates it from everything else, and which persists, at least for the time period over which it is observable. In this article, we consider the ways in which a boundary mediates the vicarious interactions between things internal and external to a system. This provides a useful way to think about biological systems, where these sorts of interactions occur at a range of scales [1,2]: the membrane of a cell acts as a boundary through which the cell communicates with its surroundings, and the same can be said of the sensory receptors and muscles that bound the nervous system. Appealing to concepts from information geometry and stochastic thermodynamics, we see that the dynamics of persistent, bounded systems may be framed as inferential processes [3]. Specifically, those states internal to a boundary appear to infer the states outside of it. An interesting consequence of this arises when we ask how we would evaluate the relative probabilities of future trajectories, given the inferences about the current state of the external world. The answer to this question takes the form of a fluctuation theorem, which says that the most probable trajectories are those with the smallest expected free energy [4]. This provides an important point of contact with planning and decision-making for those creatures who engage in temporally deep inference [5,6]. In what follows, we try to develop the links between a purely mathematical formulation of Langevin dynamics—of the kind found in the physical sciences—and descriptions of belief updating (and behaviour) found in the biological sciences.

This paper has three parts, each of which introduces a simple, but fundamental, move. The first is to partition the world into internal and external states of a system. The conditional dependencies this implies equip the internal states of the system with an information geometry for a space of (Bayesian) beliefs about the external states. More precisely, the partition means that internal states parametrize a probability density over external states. Consequently, the internal state-space has an inherent information geometry (technically, this space is a statistical manifold). The second move is to equip these beliefs with dynamics by expressing their rate of change as a gradient ascent on their non-equilibrium steady-state densities. The key consequence of this is that the dynamics of the beliefs encoded by internal states become consistent with variational inference in statistics, machine learning and theoretical biology [7]. The third move is to characterize the probability density at the level of trajectories. Through considering the reversibility of a trajectory, we associate the inferential dynamics developed in the first two sections with their thermodynamic homologues. Via the relevant fluctuation theorems, this implies a thermodynamic (i.e. energetic) characterization of inference, and lets us characterize the probability of a given path in terms of its expected free energy.

## 2. Markov blankets

### (a) Internal and external

**Figure 1.**
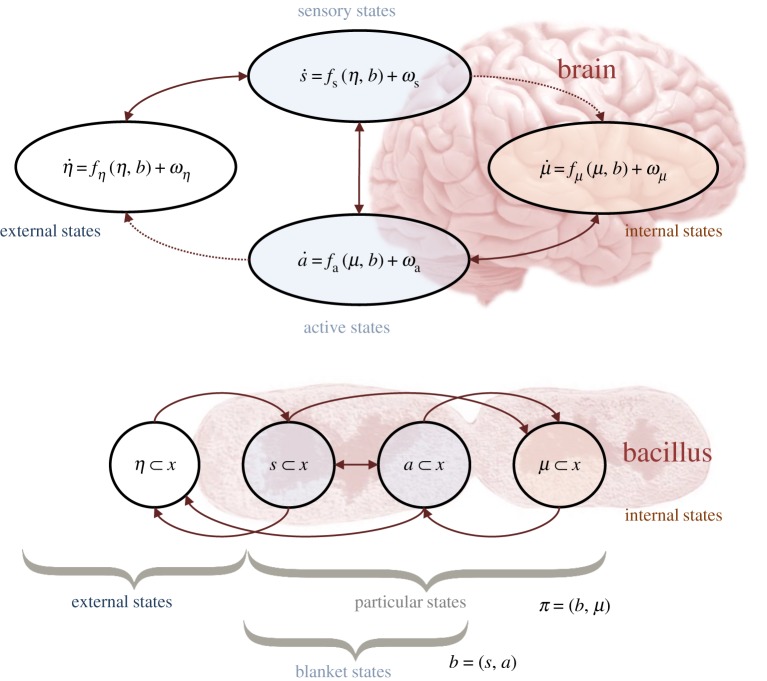
Markov blankets. This probabilistic graphical model illustrates the partition of states into internal states and hidden or external states that are separated by a Markov blanket—comprising sensory and active states. The upper panel shows this partition as it would be applied to action and perception in a brain. The ensuing self-organization of internal states then corresponds to perception, while active states couple brain states back to external states. The lower panel shows the same dependencies but rearranged so that the internal states are associated with the intracellular states of a cell, where the sensory states become the surface states or cell membrane overlying active states (e.g. the actin filaments of the cytoskeleton). Note that the only missing influences are between internal and external states—and directed influences from external (respectively, internal) to active (respectively, sensory) states. Particular states constitute a particle; namely, blanket and internal states. The equations of motion in the upper panel follow from the conditional dependencies in equation (2.1) and the Langevin dynamics in equation (3.1). See main text for details. (Online version in colour.)

This section formalizes the idea of a boundary as a Markov blanket [8]. Put simply, a Markov blanket (*b*) is the set of states that separate the internal parts of a system (*μ*) from its surroundings (*η*)—see figure 1 for two intuitive examples of this. If the system we were interested in were a brain, internal (neural) states are statistically insulated from external objects by sensory receptors and muscles. If instead we were interested in a bacillus, the cell membrane and actin filaments segregate intracellular from extracellular variables. Formally, this is a statement of conditional independence. Given knowledge of the blanket, the internal and external states of a system are conditionally independent of one another:

2.1 η⊥μ|b⇔p(η,μ|b)=p(η|b)p(μ|b).

Another way to phrase this is that any influence the external and internal states have on one another is mediated via the Markov blanket. The first step towards unpacking the consequences of this is to note that the right-hand side of equation (2.1) tells us that every blanket state is associated with a most likely internal state and a most likely external state. Pursuing the example of the brain, any given combination of retinal activity and oculomotor angle (blanket states) will be associated with a most probable object location (external state) and pattern of neural activity in the visual cortices (internal state).

2.2 $$\begin{array}{rl} \eta (b) & \;\overset{\Delta }{=}\;\underset{\eta }{\arg\max}p(\eta |b)\\ \mu (b) & \;\overset{\Delta }{=}\;\underset{\mu }{\arg\max}p(\mu |b). \end{array}\}$$

This expression assumes a unique maximum for each of the two probability densities. If this assumption were violated, this could be repaired by adding an additional condition to select between alternative modes (or by choosing an alternative statistic, such as the expectation). Given that each blanket state is associated with this pair, we can define a function that maps between the two:

2.3 η(b)=σ(μ(b)).

**Figure 2.**
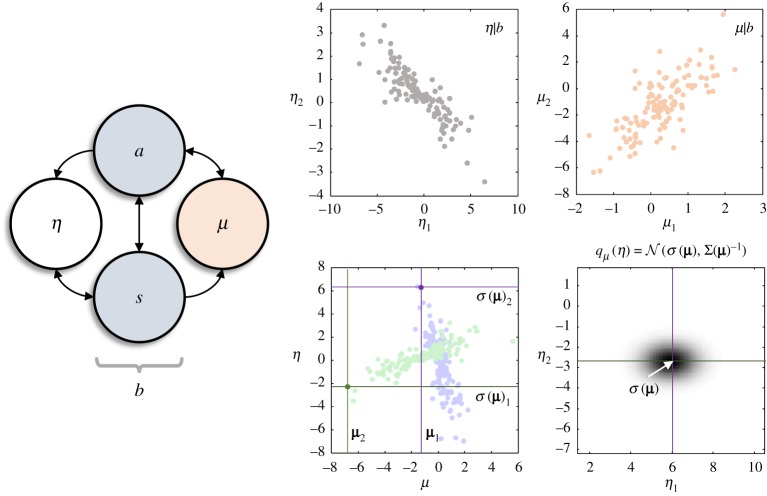
Information geometry and Markov blankets. The schematic on the left specifies the minimal set of conditional independencies required to render a set of internal states (*μ*) independent of external states (*η*), conditioned upon the blanket states (*b*). These independencies come in two flavours: as indicated by the arrows, the active states (*a*) mediate the influence of the internal states on the external states but are *not* influenced by the external states. The sensory states (*s*) mediate influences in the opposite direction and are *not* influenced by internal states. The plots on the right aim to provide some intuition for the information geometry induced by a Markov blanket. The upper two plots were created by generating (one-dimensional) blanket states from a standard normal density. For each blanket state, we generated a pair of two-dimensional internal (*μ*_1_*, μ*_2_) and external states (*η*_1_*, η*_2_). These were generated from bivariate normal densities for the conditional probabilities of the internal and external states (*p*(*μ|b*) and *p*(*η*|*b*)), where the mean and covariance were linear functions of the blanket states. As the plots show, these sufficient statistics are scattered around a low-dimensional (statistical) manifold (of the same dimension as the blanket states) embedded within the higher-dimensional external and internal state-spaces. The lower left plot shows each pair of internal and external states (blue for the first element in each, and green for the second). Selecting the most likely internal state (**μ**) for a given blanket state, we see that we can map from this to the corresponding external state. The lower right plot shows how, equipping this mean with a covariance (under the Laplace assumption), we can associate an internal state with a density (*q**_μ_***(*η*)) over external states. (Online version in colour.)

This expression tells us that, if we knew the most likely internal state of a system, we could specify the most likely external state on the other side of the Markov blanket (figure 2). A sufficient condition for this mapping to be well defined is that the mapping from *b* to **μ**(*b*) is injective. Equation (2.3) implies an information geometry that links the statistics of the two, in virtue of the boundary that separates them.

### (b) Information geometry

The central idea that underwrites information geometry [9] is that we can define a space of parameters (a statistical manifold), where each point in that space corresponds to a probability density (e.g. the expectation and variance of a normal density). For a comprehensive introduction to this field, see [10]. In brief, this leverages methods from differential geometry to characterize a statistical manifold. Central to this characterization is the notion of information length, which requires that we can define an inner product on this manifold. Appealing to the Laplace assumption [11], the most likely internal states, given blanket states, parametrize a family of (normal) densities (*q**_μ_***) for the external states. The Laplace assumption is that the negative log probability (or surprise) is approximately quadratic in the region near the mode of the density (or, equivalently, that the probability density is Gaussian near its mode):

2.4 $$\begin{array}{rl} p(\eta |b) & \propto p(\eta ,b)\\ ℑ(\eta ,b) & \approx ℑ(\sigma (\mu ),b)+\frac{1}{2}(\eta -\sigma (\mu ))\cdot \Sigma {\left(\mu \right)}^{-1}(\eta -\sigma (\mu ))\\ & \Rightarrow p(\eta |b)\approx q_{\mu }(\eta )=N(\sigma (\mu ),\Sigma {\left(\mu \right)}^{-1})\\ \Sigma {\left(\mu \right)}^{-1} & \;\overset{\Delta }{=}\;\nabla_{\sigma \sigma }ℑ(\sigma (\mu ),b)\\ ℑ(x) & \;\overset{\Delta }{=}\;-\ln p(x). \end{array}\}$$

As both the expectation and the variance of the resulting density are functions of the (most likely) internal states, the space of internal states now specifies a space of probability densities over external states. To characterize this, we need to borrow an important concept from differential geometry. This is a ‘metric tensor’ (**g**), which equips the space with the notion of length. A common method for evaluating how far a probability density has moved is to express the KL-Divergence^1^ between the initial and final density [12]. However, this does not qualify as a measure of length, as it is asymmetric (i.e. the divergence from one density to another is not necessarily the same as that from the second to the first). A solution to this is to measure the divergence over a very small change, such that a second-order Taylor series expansion is sufficient for its characterization. This provides a symmetric measure of length [13], as the Hessian matrix of the KL-Divergence is the (Fisher) information metric:

2.5 $$\begin{array}{rl} D_{\text{KL}}[q_{\mu^{\prime }}(\eta )||q_{\mu }(\eta )] & \approx \frac{1}{2}\text{d}\mu \cdot \nabla_{\mu \mu }D_{\text{KL}}[q_{\mu^{\prime }}(\eta )||q_{\mu }(\eta )]{|}_{d\mu =0}\text{d}\mu ,\text{d}\mu \;\overset{\Delta }{=}\;\mu -\mu^{\prime }\\ \sqrt{\text{d}\mu \cdot \text{g}\text{d}\mu } & =\text{d}\ell \end{array}\}$$

Note that the Euclidean inner product is a special case of this metric, when **g** is an identity matrix (i.e. the information gain along one coordinate is independent of that along other coordinates). Given that we could write down a parametrization (*λ*) of the density over internal states—and we have seen above that the internal states parametrize beliefs about the external states—the internal states participate in two statistical manifolds, with the following metrics:

2.6 $$\begin{array}{rl} {\text{g}}_{\lambda } & =\nabla_{\lambda \lambda }D_{\text{KL}}[p_{\lambda^{\prime }}(\mu )||p_{\lambda }(\mu )]{|}_{\text{d}\lambda =0}\\ {\text{g}}_{\mu } & =\nabla_{\mu \mu }D_{\text{KL}}[q_{\mu^{\prime }}(\eta )||q_{\mu }(\eta )]{|}_{\text{d}\mu =0}. \end{array}\}$$

The key conclusion from this section is that the presence of a Markov blanket induces a dual information geometry with two metric tensors: one that describes the space of densities over the internal states (**g***_λ_*), and one that treats the internal states as points in a space of densities over external states (**g*_μ_***).

## 3. Dynamics and inference

### (a) Non-equilibrium steady state

Building upon the previous section, we now ask: If a system maintains its separation from the outside world over time, what sort of dynamics must it exhibit? To answer this question, we start with a general description of stochastic dynamics. This is a Langevin equation, describing the rate of change of some variable (*x*) in terms of a deterministic function ( *f*(*x*)), and fast fluctuations (*ω*). The fluctuations are assumed to be normally distributed, with an amplitude of 2*Γ* (a diagonal covariance matrix):

3.1 $$\begin{array}{rl} \dot{x} & =f(x)+\omega \\ E[\omega (\tau )] & =0\\ E[\omega (\tau )\omega (t)] & =2\Gamma \delta (\tau -t) \end{array}\}\Leftrightarrow \dot{p}(x)=\nabla \cdot (\Gamma \nabla -f(x))p(x).$$

The expression on the right of equation (3.1) is the Fokker–Planck equation [14,15], which provides an equivalent description of the stochastic process on the left, but in terms of the deterministic dynamics of a probability density. This may be thought of as expressing a conservation law for probability, as the rate of change of the density is equal to the (negative) divergence (∇⋅) of the probability current. This is useful in formalizing the notion that a system maintains its form over time, as we may set the rate of change of the density to zero, and find the flow that satisfies this existential condition:

3.2 $$\begin{array}{rl} & \dot{p}(x)=0\Leftrightarrow f(x)=(Q-\Gamma )\nabla ℑ(x)\\ & \nabla \cdot (Q\nabla p(x))=0. \end{array}\}$$

The above equation constitutes a general description for a system at non-equilibrium steady state [16,17]. This has two components (consistent with a Helmholtz decomposition), each of which depends upon the gradient of the negative log probability (or surprise) of the steady-state density. The first flow component is solenoidal (divergence-free) and involves a flow around the contours of the surprise. This arises from the *Q*-matrix, often assumed to be skew-symmetric (i.e. *Q* = −*Q*^T^). The second part depends upon the amplitude of fluctuations (*Γ*) and performs a gradient descent on surprise (negative log probability). Intuitively, the greater the amplitude of the fluctuations, the greater the velocity required to prevent dispersion due to random fluctuations. In the following, we partition *x* into internal, external and blanket states. To keep things simple, we assume a block diagonal form for *Q*. This extends the concept of a Markov blanket to a dynamical setting such that, in addition to the current values of the internal and external states being conditionally independent of one another, their rates of change are also uncoupled.

### (b) Bayesian mechanics

Taking equation (3.2) as our starting point, we can now examine the dynamics of a system with a Markov blanket. We focus upon those states that are not directly influenced by the external states, which include the internal states and a subset of the blanket states that we refer to as ‘active’ states (*a*) that mediate the influence of the internal states on the external states (as opposed to ‘sensory’ states that mediate the opposite influence). First, using a (Moore–Penrose) pseudoinverse,^2^ we can use the chain rule to express the rate of change of the most likely internal states (i.e. the population dynamics [18]) in terms of the flow of the most likely external states:

3.3 $$\begin{array}{rl} & \dot{\eta }(b)=\nabla_{\mu }\sigma \dot{\mu }(b)\\ & \Rightarrow \dot{\mu }(b)={\left(\nabla_{\mu }\sigma \right)}^{-}\dot{\eta }(b). \end{array}\}$$

A similar application of the chain rule lets us express the gradient of the surprise with respect to external states as a gradient with respect to internal states:

3.4 $$\begin{array}{rl} & \nabla_{\mu }ℑ(\sigma (\mu ),b)=\nabla_{\mu }\sigma \nabla_{\eta }ℑ(\eta ,b)\\ & \Rightarrow {\left(\nabla_{\mu }\sigma \right)}^{-}\nabla_{\mu }ℑ(\sigma (\mu ),b)=\nabla_{\eta }ℑ(\eta ,b). \end{array}\}$$

This lets us write the flow of external states as a function of the gradient of internal states (using equation (3.2)):

3.5 $$\begin{array}{rl} \dot{\eta }(b) & =(Q_{\eta \eta }-\Gamma_{\eta \eta })\nabla_{\eta }ℑ(\eta ,b)\\ & =(Q_{\eta \eta }-\Gamma_{\eta \eta }){\left(\nabla_{\mu }\sigma \right)}^{-}\nabla_{\mu }ℑ(\sigma (\mu ),b). \end{array}$$

Substituting this into equation (3.3) then gives

3.6 $$\begin{array}{rl} \dot{\mu }(b) & =-\Gamma_{\sigma \sigma }\nabla_{\mu }ℑ(\sigma (\mu ),b)\\ \Gamma_{\sigma \sigma } & \;\overset{\Delta }{=}\;{\left(\nabla_{\mu }\sigma \right)}^{-}\Gamma_{\eta \eta }{\left(\nabla_{\mu }\sigma \right)}^{-}. \end{array}\}$$

**Figure 3.**
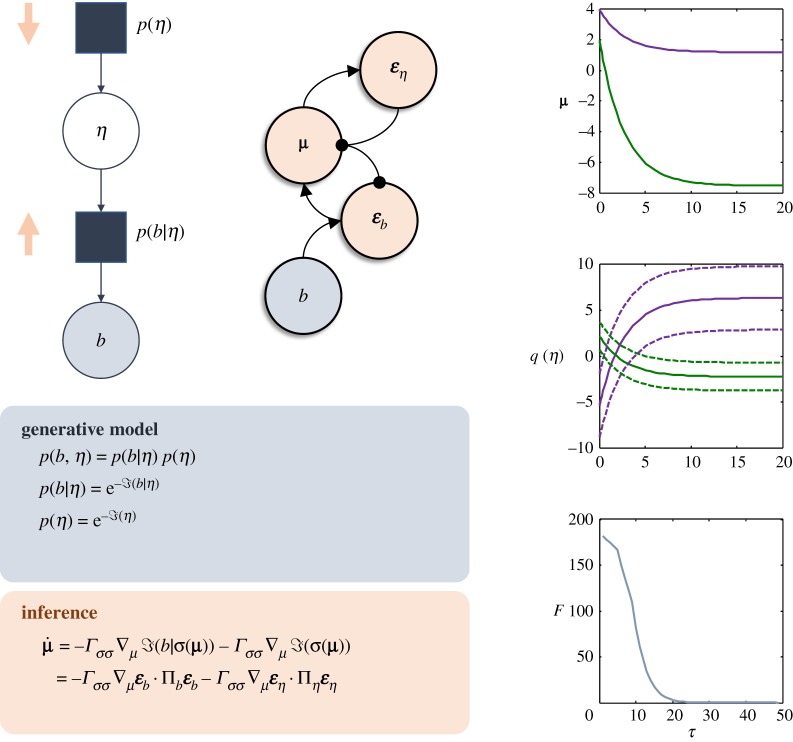
Inferential dynamics and generative models. As outlined in the main text, the dynamics of the internal states of a system minimize the joint surprise of external and blanket states. Interpreting this joint density as a generative model, we can interpret gradient flows at non-equilibrium steady state in terms of variational inference, as shown here. The upper left graph illustrates the interpretation of the surprise in terms of a generative model (using a factor graph formalism) with a prior and a likelihood (squares). In the setting of variational inference, the dynamics that solve this inference problem may be framed as message passing, where the likelihood and prior each contribute local messages (pink arrows) that are combined to evaluate a (marginal) posterior probability. This provides an important point of contact with concepts like predictive coding [20] in theoretical neurobiology, as illustrated in the pink panel, and in the graphic on the right of the factor graph. This formulation uses the second-order Taylor expansion of surprise and interprets the difference between the predicted and observed blanket states as a prediction error (***ε****_b_*)—similarly for the difference between the prior and posterior expectations for the external states (***ε****_η_*). These errors drive updates in the internal states representing expected external states. This leads to a process of prediction error minimization. The plots on the right illustrate the dynamics of equations (3.6) and (3.8). If we initialize the expected internal states away from their mode under the steady state density, we see that they return to this. As they do so, the beliefs they represent become consistent with those shown in figure 2, and the free energy (*F*) difference from its steady-state value returns to zero. For readers who wish to gain an intuition for the dynamics of Markov-blanketed systems, a series of numerical demonstrations [21] may be accessed through academic software available at http://www.fil.ion.ucl.ac.uk/spm/software/. Typing DEM at the Matlab prompt will invoke a graphical user interface through which a range of simulations may be accessed and customized. This includes examples of practical applications of Bayesian mechanics in numerous domains. See also https://tejparr.github.io/Physics/Slides%20main.htm for a graphical introduction to these topics. (Online version in colour.)

This result has an interesting interpretation in the setting of statistical inference. Interpreting surprise in terms of a statistical (i.e. generative) model [19], as expressed in figure 3, equation (3.6) acquires the interpretation of a *maximum a posteriori* (MAP) inference scheme. Remembering that the internal states are associated not only with a most likely external state, but with a full probability density (equation (2.4)), we can go further and associate the dynamics of equation (3.6) with variational Bayesian inference [7]. Variational Bayes rests upon the minimization of a quantity known as ‘free energy’, which is an upper bound on the surprise (i.e. negative log probability) of blanket states (this is equivalent to maximizing an evidence lower bound, or ‘ELBO’):

3.7 $$\begin{array}{rl} F(\mu ,b) & \;\overset{\Delta }{=}\;ℑ(b)+D_{\text{KL}}[q_{\mu }(\eta )||p(\eta |b)]\\ & =E_{q_{\mu }}[ℑ(\eta ,b)+\ln q_{\mu }(\eta )]\\ & \approx ℑ(\sigma (\mu ),b)+tr[\Sigma (\mu )\underset{\Sigma {\left(\mu \right)}^{-1}}{\underbrace{\nabla_{\sigma \sigma }ℑ(\Sigma (\mu ),b)}}]-\frac{1}{2}\ln|\Sigma (\mu )|. \end{array}$$

The final line makes the same Laplace assumption employed in equation (2.4). This means that the second and third terms in the last equality are constant with respect to the mode, as the curvature of a quadratic function is constant. As such, we can rewrite equation (3.6) as follows:

3.8 $$\dot{\mu }(b)=-\Gamma_{\sigma \sigma }\nabla_{\mu }F(\mu ,b).$$

From the first line of equation (3.7), this means we can interpret (the expected flow of) internal states as minimizing a bound on the surprise of the blanket states. Interestingly, a similar interpretation applies to the active states (constituents of the Markov blanket). As the active states of the Markov blanket do not depend upon the external states, they will perform a gradient descent on the joint surprise of internal and blanket states:

3.9 $$\begin{array}{rl} \dot{a}(\mu ) & =(Q_{aa}-\Gamma_{aa})\nabla_{a}ℑ(\mu ,b)\\ & =(Q_{aa}-\Gamma_{aa})\nabla_{a}ℑ(\sigma (\mu ),b)\\ & =(Q_{aa}-\Gamma_{aa})\nabla_{a}F(\mu ,b). \end{array}$$

The final line of equation (3.9) summarizes the conclusion of this section. Both internal and active states minimize variational free energy, and therefore the surprise of blanket states. The latter is known in statistics as negative (Bayesian) model evidence. This implies that Markov-blanketed systems with a non-equilibrium steady state may be thought of as ‘self-evidencing’ [22]. From a physiologist's perspective, this is simply a statement of homeostasis [23], where (active) effectors correct any deviation of sensory states from normal physiological ranges.

An interesting aspect of the analysis presented in this section is that it does not commit to a spatial or temporal scale. This is important, as it means that the interpretation of the dynamics of internal states depends upon the scale at which we identify their Markov blanket. Typically, this depends upon the system of interest, but it is important to recognize that we can recursively subdivide (or combine) Markov-blanketed systems and select alternative levels of description. For example, at the level of a population, blanket states are characteristics of those individuals who intersect with other populations. The internal states of one population will appear (on average) to infer those of other populations. We could, however, select an individual within this population as our object of study. The internal states of this individual will appear to make inferences about those in the original population who, in virtue of our taking an alternative perspective, have gone from being internal states (performing inference) to external states (being inferred). We could go further, and select an organ, tissue, cell or molecule as our blanketed system. At each level, the content of the inference implicit in internal state dynamics will change, but will still be subject to the Bayesian mechanics outlined above.

## 4. Stochastic thermodynamics

### (a) Path integrals and reversibility

How do we go from the description above to concepts like ‘heat’ that are central in characterizing the energetics of inference? The key to this is to move from thinking about a density over a particle's current location to quantifying the probability of it having followed a given path. This is given by the following (Stratonovich^3^) path integral [26]:

4.1 $$ℑ(x[\tau ])=\frac{1}{4\Gamma }\int_{o}^{t}(\dot{x}\cdot \dot{x}-2\dot{x}\cdot f+f\cdot f+2\Gamma \nabla \cdot f)\;\text{d}\tau .$$

If we compare this to the probability associated with the same path, but in the opposite direction (as if we had reversed time), we obtain:

4.2 $$ℑ(x[t-\tau ])-ℑ(x[\tau ])=\frac{1}{\Gamma }\int_{0}^{t}\dot{x}\cdot f\text{d}\tau =\frac{1}{\Gamma }\text{q}.$$

This equation says that the amount of heat dissipated (**q**) along a given path is an expression of how surprising it would be to observe a system following the same path backwards relative to forwards. Substituting equation (3.2) into this (ignoring solenoidal flow) gives

4.3 $$\text{q}=-\Gamma \int_{0}^{t}\dot{x}\cdot \nabla ℑ(x)\text{d}\tau =-\Gamma \int_{0}^{t}\dot{ℑ}(x)\text{d}\tau =-\Gamma \Delta ℑ(x)=-k_{\text{B}}T\Delta ℑ(x).$$

The final equality decomposes the amplitude of random fluctuations into Boltzmann's constant and the temperature of the system, ensuring consistency of units. Considering a trajectory for expected external states, this expresses the heat dissipated by a change in free energy:

4.4 $$\text{q}=-k_{\text{B}}T\Delta ℑ(\sigma (\mu ),b)\approx -k_{\text{B}}T\Delta F(\mu ,b).$$

The approximate equality again rests upon the assumption that, as long as we do not move far from the mode, the Hessian of the surprise does not change. This allows us to equate changes in free energy with changes in the joint surprise of internal and blanket states, ignoring any changes in the entropy of the variational density (*q*(*η*)). The result is a limiting case of Jarzynski's inequality [27], relating a change in free energy (i.e. an inference) to the heat dissipated in the process.

### (b) Fluctuation theorems

The use of time-reversal above, and the temporal asymmetry that gives rise to heat, is one example of the use of ‘conjugate’ dynamics (†). More generally, this concept may be exploited to derive a set of results known as the ‘fluctuation theorems' [28]. The idea here is that certain scalar functionals (*S*) of a trajectory will have the same magnitude under an alternative (e.g. time-reversed) trajectory. For example, as indicated by equation (4.2), heat has the same magnitude (but reversed sign) under a time-reversed protocol. Putting this more formally, we start with a functional consistent with the following:

4.5 $$S^{†}(x^{†}[\tau ])=\varepsilon S(x[\tau ]),\;\varepsilon =\pm 1.$$

For an arbitrary function (*g*(*S*)), this may be used to derive the master fluctuation theorem [28]:

4.6 $$\begin{array}{rl} E_{p(x[\tau ])}[g(\varepsilon S)] & =\int g(\varepsilon S)p(x[\tau ])\text{d}x[\tau ]\\ & =\int g(S^{†})p(x^{†}[\tau ])\frac{p(x[\tau ])}{p(x^{†}[\tau ])}\text{d}x^{†}[\tau ]=E_{p(x^{†}[\tau ])}\left[g(S^{†})e^{ℑ(x^{†}[\tau ])-ℑ(x[\tau ])}\right]. \end{array}$$

Under different choices for *g*(*S*), or different choices of conjugate dynamics, equation (4.6) can be used to derive the fluctuation theorems that underwrite stochastic thermodynamics. For interested readers, a comprehensive treatment of this subject is given by Seifert [28]. Here, we focus on a fluctuation theorem that arises in virtue of the dual information geometry of equation (2.6). If we choose *g*(*S*) = 1, and set the conjugate dynamics to be those given knowledge of the current (average) internal state, we obtain an integral fluctuation theorem that provides an upper bound for the expected surprise (or entropy) of a future trajectory:

4.7 $$\begin{array}{rl} p(x^{†}[\tau ]) & =q(x[\tau ])=p(x[\tau ]|\mu )\\ & \Rightarrow E_{q}\left[\ln\frac{q(\eta [\tau ]|\pi [\tau ])}{p(\eta [\tau ],\pi [\tau ])}-ℑ(\pi [\tau ]|\mu )\right]\geq 0. \end{array}$$

We have used the notation *π* = (*μ*,*b*), to group the internal states and their blanket. Together, these are referred to as *particular* states. There is an interesting connection between equation (4.7) and the information length of a trajectory [29,30]. Once non-equilibrium steady state has been achieved, there is no further increase in information length. This means that, if the information length between ***μ*** and the most likely value for the internal states under non-equilibrium steady state were zero, the inequality above would be an equality, as the two densities would be identical. Rearranging equation (4.7), we can express an upper bound (*G*) on the expected surprise associated with a given trajectory (where the tightness of the bound depends upon the information length):

4.8 $$\begin{array}{rl} G(\mu ) & \geq E_{q}[ℑ(\pi [\tau ]|\mu )]=H[q(\pi [\tau ])]\\ G(\mu ) & \;\overset{\Delta }{=}\;E_{q}[ℑ(\eta [\tau ],\pi [\tau ])+\ln q(\eta [\tau ]|,\pi [\tau ])]\\ & =\underset{\text{Risk}}{\underbrace{E_{q}[D_{\text{KL}}[q(\eta [\tau ]|\pi [\tau ])||p(\eta [\tau ])]}}+\underset{\text{Ambiguity}}{\underbrace{E_{q}[H[p(\pi [\tau ]|\eta [\tau ])]]}}. \end{array}\}$$

This implies that those future dynamics (i.e. choice of *q*(*π*[*τ*])) that would be least surprising (on average) given current internal states are those that have the lowest risk (i.e. where the predicted trajectory of the external states shows minimal divergence from those at steady state), while also minimizing the ambiguity of the association between external states and particular states. The interesting thing about this result is that the two quantities that comprise expected free energy, namely risk and ambiguity, are exactly the same quantities found in economics, decision theory and cognitive neuroscience. In short, many apparently purposeful behaviours can be cast in terms of minimizing risk (i.e. the KL-Divergence between predicted and *a priori* predictions of outcomes in the future) and ambiguity (i.e. the expected uncertainty about particular states, given external states of the world). We conclude by considering to what extent this anthropomorphic interpretation is licensed by the underlying physics.

## 5. Discussion

In the above, we started from the simple, but fundamental, condition that a system must remain separable from its environment for an appreciable length of time [31]. On unpacking this notion—using concepts from information geometry and thermodynamics—we found that the states internal to a Markov blanket look as if they perform variational Bayesian inference, optimizing posterior beliefs about the external world. In fact, both active and internal states (on average) minimize an upper bound on surprise. This means that Markov-blanketed systems make their world less surprising in two ways. They change their beliefs to make them more consistent with sensory data and change their sensory data to make them more consistent with their beliefs.

The sort of inference (or generalized synchrony [32,33]) we have described here is very simple, where we have grouped all external states together. However, the broad distinction we have drawn between internal and external states could be nuanced by subdividing the external states into other systems with Markov blankets. From the perspective of the internal states, this leads to a more interesting inference problem, with a more complex implicit generative model. It may be that the distinction between the sorts of systems we generally think of as engaging in cognitive, inferential, dynamics [34] and simpler systems [35] rests upon the level of sophistication of the generative models that best describe their dynamics or gradient flows [36]. For example, if we distinguish between states as positions, velocities, accelerations, etc., of external states the ensuing dynamics of internal states become consistent with temporally deep inference, and generalized Bayesian filtering [37] (a special case of which is an extended Kalman–Bucy filter [38]).

Things become more interesting when we think not just about probabilities of states, but of their trajectories [39]. This provides an important connection to concepts from thermodynamics, including the concept of heat. Crucially, this quantity may be thought of as an expression of the differences in the probability of a trajectory under different sorts of (conjugate) dynamics. This is the idea that underwrites the fluctuation theorems of stochastic thermodynamics, each of which depends upon alternative choices for the conjugate dynamics. Bringing the information geometry implied by a Markov blanket to bear on this, we can express an integral fluctuation theorem that depends upon the beliefs implied by a system's internal states. This tells us that the trajectories that are expected to be least surprising are those with the lowest expected free energy, an idea that has been exploited to reproduce a range of behaviours in computational neuroscience (e.g. saccadic eye movements in scene construction that seek out the least ambiguous sensory states [40]).

The decomposition of expected free energy into ‘risk’ and ‘ambiguity’ offers some intuition as to what this means. Suppose we have a Markov-blanketed creature whose generative model is sufficiently sophisticated that it can draw inferences about the way in which it will act. The alternative trajectories it could take may be scored by the risk and ambiguity associated with these trajectories. Anthropomorphizing, we can think of this creature as behaving like a scientist; inferring a course of action (i.e. series of experiments) that will provide the most informative (least ambiguous) data, facilitating more precise inferences about external states of the world. However, this exploratory behaviour is not the whole story. Not only does this creature pursue those trajectories that afford uncertainty reduction [41,42], it also minimizes the divergence (risk) between anticipated external states, and those consistent with non-equilibrium steady state. Interpreting the latter as ‘preferences’, in the sense that the most likely trajectories tend towards these, we are left with a creature who acts like a ‘crooked’ scientist [43], seeking out those data that are most informative, with a bias towards those that comply with its prior beliefs.

## 6. Conclusion

This paper outlines some of the key relationships between non-equilibrium dynamics, inference and thermodynamics. These relationships rest upon partitioning the world into those things that are internal or external to a boundary, known as a Markov blanket. The blanket induces a dual information geometry that lets us treat internal states as if they represent densities over external states. When equipped with dynamics, the average internal states appear to engage in variational inference. Moving to a trajectory-level characterization, we can draw from the tools of stochastic thermodynamics to relate inference to the heat it dissipates, and develop an integral fluctuation theorem that draws from the dual information geometric perspective above. This provides an (expected free energy) bound on the expected surprise (entropy) of future trajectories. Ultimately, the drive towards explorative and exploitative behaviours that this implies offers a principled way of writing down the prior beliefs of creatures who engage in planning as (active) inference.

## Footnotes

1 The KL-Divergence is a weighted average of the log ratio of two probability densities; also known as a relative entropy or information gain.

2 Assuming ${\left(\nabla_{\mu }\sigma \right)}^{-}\nabla_{\mu }\sigma =I$.

3 This expression for the path integral arises from the Stratonovich interpretation of a stochastic differential equation. However, it is worth noting that other interpretations are widely used, and may be more appropriate in many settings. For details, see [24,25].

## Data accessibility

This article has no additional data.
